# A General Primer for Data Harmonization

**DOI:** 10.1038/s41597-024-02956-3

**Published:** 2024-01-31

**Authors:** Cindy Cheng, Luca Messerschmidt, Isaac Bravo, Marco Waldbauer, Rohan Bhavikatti, Caress Schenk, Vanja Grujic, Tim Model, Robert Kubinec, Joan Barceló

**Affiliations:** 1 Hochschule für Politik, Technical University of Munich, Richard-Wagner Str. 1, Munich, 80333 Bavaria Germany; 2 School of Humanities and Social Sciences, Nazarbayev University, Kabanbay Batry Ave., 53, Astana, 010000 Kazakhstan; 3 Faculty of Law, University of Brasilia, Campus Universitário Darcy Ribeiro Asa Norte, Brasília, 10587 Brazil; 4 Delve, 2225 3rd St, San Francisco, 94107 California USA; 5 Division of Social Science, New York University Abu Dhabi, Social Science Building (A5), Abu Dhabi, 129188 United Arab Emirates

**Keywords:** Databases, Research management

## Abstract

Data harmonization is an important method for combining or transforming data. To date however, articles about data harmonization are field-specific and highly technical, making it difficult for researchers to derive general principles for how to engage in and contextualize data harmonization efforts. This commentary provides a primer on the tradeoffs inherent in data harmonization for researchers who are considering undertaking such efforts or seek to evaluate the quality of existing ones. We derive this guidance from the extant literature and our own experience in harmonizing data for the emergent and important new field of COVID-19 public health and safety measures (PHSM).

## Introduction

Unprecedented technological advancements in information technology have ushered in a data science revolution, allowing scholars, companies and policy makers to conduct analyses at a scale, speed and granularity previously unimaginable^1,2^. However as Elshawi *et al*.^3^ note, “in practice, big data science lives and dies by the data. It mainly rests on the availability of massive datasets, of that there can be no doubt.” From fields as varied as socio-economics^2,4–6^ to ecology^7^ and the ‘Internet of Things’^8^, data scientists report the lack of big data itself is a major bottleneck in using big data tools. Increasingly, data scientists must first sort through heterogeneous, incongruent, and fragmented datasets before any analyses can be conducted^9–15^. Such problems with data availability are often exacerbated in emergency situations where real time analyses are often stymied by unevenly documented or unclean data^16^[pg. 358].

Data harmonization, the practice of combining different datasets to maximize their comparability or compatibility, has become an increasingly common method for dealing with these data roadblocks. However, because data harmonization often entails untangling how complex data can be made to fit together, unsurprisingly, existing research on it has been both highly technical and field-specific^12,17–23^. Correspondingly, researchers interested in pursuing their own data harmonization or in evaluating the work of others currently lack a general primer to help them think through the tradeoffs of pursuing data harmonization in the first place. General guidance is sorely needed given that data harmonization should not be understood as merely a technical exercise in combining datasets. It also entails theoretical challenges in parsing and reconciling how concepts are understood and operationalized across different datasets as well as a logistical challenge in dealing with different data partners and stakeholders, issues that are common across fields. Moreover, the need for this kind of overview will only grow given the increasing importance of data harmonization to big data science. To address this gap, this commentary presents a general primer which (i) provides a basis for understanding the various dimensions of data harmonization (ii) encourages researchers to think through its various challenges, complexities and tradeoffs.

To develop this primer, we take a broad overview of existing data harmonization efforts from both the natural and social sciences and draw on our experience in harmonizing data on a pressing and emergent new research field, COVID-19 public health and safety measures (PHSM). With regards to the existing literature, we reviewed the most recent and/or highly cited articles on data harmonization in a wide variety of fields, including geography (e.g. soil maps^24^, land-use maps^25^), health (e.g. epidemiology^20^, medical imaging^26^, electronic medical records^27^), social sciences (e.g. economic indicators^4,5^, demography^28^), ultimately resulting in around 100 studies. By considering lessons for data harmonization across a broad number of subject areas, we both distill issues common to many of them while further elucidating perspectives that data scientists may otherwise not come across if narrowly focused within their own field of study. Meanwhile, our own data harmonization effort has entailed harmonizing data on government responses to the COVID-19 pandemic across 8 disparate and complex datasets, which we elaborate on more fully in Cheng *et al*.^29^. To the extent that examples from a specific data harmonization effort can be helpful for understanding the process overall, we draw heavily from our experience here. We hope that both the data scientist seeking to bolster the rigor of their data harmonization efforts as well as the data scientist judging the quality of other’s work will be able to make use of the issues raised here for their respective ends.

In what follows, in the first half of the commentary, we first describe what data harmonization is both at the conceptual level and procedural level. With regards to the former, given the complexity inherent in harmonizing data we introduce what data harmonization is both by definition as well as in contrast to other similar methods of combining data. With regards to the latter, we outline different types of data harmonization and provide a general overview of how it can be implemented. Having established this foundation for understanding what data harmonization is, we then spend the second half of the commentary detailing the different tradeoffs inherent to harmonizing data relative to original data collection.

## What is data harmonization?

This section first addresses how data harmonization can be defined and conceptualized on its own and in relation to similar concepts. It then provides an overview as to how data harmonization can be achieved, both in terms of its different archetypes as well as in terms of the various broad steps the process entails.

### How is data harmonization defined?

Data harmonization is the practice of “reconciling various types, levels and sources of data in formats that are compatible and comparable, and thus useful for better decision-making”^30^[pg. 360]^31^ or analysis. For example, in our effort to harmonize data on COVID-19 government policies (like lockdowns and travel bans) from 8 separate datasets, we had to reconcile heterogeneity in the data for a number of dimensions, including the definitions of policies themselves (e.g. restrictions to move across borders were defined differently across datasets). Note, data harmonization can be done not only with respect to reconciling different operationalizations of similar concepts (like in the example of COVID-19 government policies given above), but also with respect to reconciling more mundane measurement differences (e.g. when the same device produces different measurements or when different devices are used to measure identical or similar subjects). More generally, to create a harmonized dataset, data harmonization can be understood as resolving heterogeneity along at least three dimensions^32^[pg. 9-10]:

- **Syntax** (i.e. data format): Data can come in a variety of technical formats (e.g. .csv, JSON, HTML) that can require additional processing before the data can be harmonized.
- **Structure** (i.e. conceptual schema): This refers to how different variables relate to each other within a dataset; these can vary widely across datasets. On one end of the spectrum is structured data, which are highly organized and formatted (e.g. data tables), to unstructured data with little or no fixed format (e.g. raw text, images). Different datasets can have large sources of variation not only across types of data structures but within them. Structured data, for example, can come in many forms: while most datasets on COVID-19 PHSM are structured as event data where each row represents a given policy event, each row of the Oxford COVID-19 Government Response Tracker (OxCGRT)^33^ panel data represents a country-day and a given policy event can span across multiple rows. If a lockdown was implemented in country A from March 1 to March 3, 2020, the event dataset format captures this information in one row of data to correspond to the policy event, with one variable to capture the event start date and a second variable to capture the event end date. In the panel data format, this event is captured across three rows of data, one for each day, with one variable capturing each day of the month.
- **Semantics** (i.e. intended meaning of words): A close reading of what a given variable is intended to measure is necessary in order to properly harmonize variables across datasets. For instance, use of the same terminology does not guarantee that different datasets are measuring the same concept. To provide an illustrative example, while a number of datasets may purport to measure the incidence of a disease across *young adults*, one dataset may define young adults as people from the ages of *18 to 25* while another may define it as ranging from *18 to 30*. Correspondingly, it is also possible that different datasets may use distinct terminology but nevertheless be measuring the same concept. For example, one dataset which purports to measure the incidence of disease across *young adults* may be the same as another which purports to measure the incidence of disease across *teenagers* if ultimately, both datasets in fact capture this information for people ages 13 to 18.

Regardless of how harmonization is conceptualized it can be broadly understood as stringent or flexible^34^. Stringent harmonization refers to the use of identical measures and procedures across studies. Meanwhile flexible harmonization ensures that different datasets are, though not necessarily identical, inferentially equivalent and ultimately transformed into a common format. Often different dimensions of data harmonization can be considered as being stringently or flexibly harmonized. For example, our ongoing effort to harmonize 8 datasets on COVID-19 government responses can be understood stringent insofar as all observations were collected using the same ontology and protocol developed by the CoronaNet Research Project^29^. However, the raw sources on the government policies were identified using procedures and guidance that varied from dataset to dataset, and the act of pooling these raw sources can be understood as an instance of flexible harmonization.

### How is data harmonization different from other ways to combine data?

There are a plethora of ways that data can be manipulated or analyzed. In the following, we elucidate how data harmonization is distinct from other methods it is commonly associated with: data integration, data standardization and meta-analysis, to help clarify what it is and is not.

First, data harmonization is distinct from data integration, also known as data linkage^35^, in that (successful) data harmonization results in a dataset that follows a unique, cohesive ontology or taxonomy derived from conceptually similar datasets (e.g. combining multiple datasets on COVID-19 PHSM into one dataset on COVID-19 PHSM). Meanwhile data integration results in a multidimensional dataset made from conceptually different datasets (e.g. combining datasets on COVID-19 PHSM, COVID-19 deaths, and GDP together into e.g. the PERISCOPE Data Atlas^36–38^. Note however, that while data harmonization results in a single taxonomy or ontology that can be used across different datasets, the harmonized data itself may be constructed as a single dataset or remain dispersed across multiple datasets depending on the various ethical, legal, methodological or logistical factors at play^39^.

Standardization meanwhile, aims to unify data using a uniform methodology. How harmonization is understood to relate to standardization can be field or domain specific, with some asserting it to be a difference of degree, while others asserting it to be a difference of kind. With regards to the former, standardization can be understood to be the most extreme form of stringent harmonization possible insofar as all potential dimensions of the data (i.e. structure, syntax, semantics) are made to be identical and often held up to be the primary reference point for a given domain. A softer interpretation under this perspective is that standardization is one step on the way to harmonization given that differences among datasets must eventually be resolved into a common understanding, regardless of whether this understanding is subsequently regarded as being the primary reference point. With regards to the latter, standardization is seen as qualitatively different from harmonization insofar as harmonization is understood as not “impos[ing] a single methodology or norm, but rather seeks to find ways of integrating or making ‘an agreeable effect’ from information gathered through disparate methodologies.”^40^. Under this perspective, while standardized data can be considered as harmonized, harmonized data is not necessarily standardized^41,42^. Both understandings are widely used and to avoid confusion, definitions for these terms should be provided accordingly. We employ the ‘difference of degree’ definition in our commentary.

Finally, meta analyses, also known as aggregate data meta analysis^43^[pg. 70], is a methodological strategy for synthesizing different research studies which has become increasingly used over the past four decades across a variety of fields^44,45^. In brief, it restricts itself to combining information on summary statistics of different datasets rather than combining the underlying data itself^46^[pg. 21]. Note that, mega-analysis, also known in some fields as individual participant data (IPD) meta-analysis, entails synthesizing information by pooling the raw data, and can be understood as a form of data harmonization. In one stage IPD, analyses are done on one pooled and harmonized dataset, whereas in two stage IPD, analysis are done on each dataset separately before being pooled^43^. Overall, while some studies suggest that mega-analyses/data harmonization can yield more precise results than meta-analyses^12,47^, others find that they can substantively be quite similar^48,49^ and that differences between the two can largely be explained by differences in modelling assumptions as opposed to intrinsic reasons^50^. In fields where meta-analyses are constrained to only peer-reviewed published works however, the underlying data may be highly biased both from the publication process itself, which generally biases against publishing null findings, and selection bias on the part of the original authors, who may only present the subset of data which most strongly supports their hypotheses^51^.

### How can data harmonization be implemented?

This section provides a general typology followed by a step-by-step overview of the harmonization processes in order to provide researchers with a procedural perspective of how data harmonization can be achieved. In our review of the available literature, even guidelines which strive to be general are in fact targeted toward specific fields e.g. epidemiology^20^ or medicine^52^. In cases when such guidelines can be applied to other fields, the degree to which knowledge of jargon or field-specific information is needed to understand them may be a barrier toward using them. Meanwhile, insofar as different harmonization efforts have different goals, there may be any number of different ways to pursue data harmonization for the same set of underlying data^53^, which can be difficult to ascertain without access to more general guidance.

While field specific guidelines^24,28,54–58^ are still important to consult given that implementing data harmonization can be highly contingent on the nuances and idiosyncrasies of the original datasets being harmonized, our general overview can hopefully provide better context for understanding and evaluating the data harmonization process. Moreover, to the extent that a given methodological decision can be made with full knowledge of possible alternative choices, the ultimate quality of harmonized data can ideally be raised in comparison to having only partial knowledge.

### Types of Implementation

Both methodological and theoretical considerations can heavily influence how datasets are ultimately harmonized. In terms of methodological considerations, data harmonization can take place (i) after the data has already been collected, commonly known as retrospective harmonization (also known as ex-post harmonization or output harmonization)^6,20^) or (ii) before the data has been collected, commonly known as prospective harmonization (also known as ex-ante harmonization or input harmonization^6^). The characterization of retrospective and prospective harmonization presented here should be considered as archetypes, with the understanding that actual harmonization efforts often lie on a continuum between the two^51^. Meanwhile, in terms of theoretical choices, which concepts researchers wish to operationalize and the feasibility of doing so across different datasets profoundly affects which variables are ultimately harmonized and how. We discuss each of these considerations in turn.

#### Retrospective data harmonization

Retrospective harmonization refers to harmonization of already collected datasets. Our ongoing efforts to harmonize 8 COVID-19 PHSM datasets can be considered a case of retrospective data harmonization because all constituent datasets were collected prior to the harmonization process^29^. In some circumstances, original data collection is not possible and only retrospective harmonization is feasible. This is canonically true whenever researchers wish to analyze past events or behaviours^25^. For instance, those seeking to analyze survey data for a past time frame must rely on previous surveys, if any exist, and work with the set of questions asked at the time^59–66^. In other cases, while original data collection may theoretically be possible, the time impermanence of primary sources may render it unfeasible to fully implement. For instance, while harmonizing mobile phone usage presents a promising avenue for analyzing mobility patterns, the rapid pace at which new providers arise and the extent to which users switch between services means that the accessibility or availability of this data are often difficult to maintain^67^.

#### Prospective data harmonization

Prospective harmonization is a distinct form of original data collection where research methodologies are harmonized before (at least some) data collection takes place. While in its purest form, research methodologies are harmonized before any data collection takes place, data harmonization can still be considered prospective if data scientists collect data derived from methodologies used on already collected data.

Though prima facie, prospective data harmonization would appear to be strictly dominant over both original data collection and retrospective data harmonization, its disadvantages can be substantial. First, it can be challenging to implement because it requires agreement on standardized measures among different researchers who likely have diverse research goals. These challenges are exacerbated when standardized measures must be created contemporaneously^68^. Moreover, if a given dataset already exists and possesses a substantial history and organizational support, it may require tremendous effort to overcome institutional resistance to coordinate different stakeholders around a new methodology^69^. Furthermore, there is no guarantee that the resulting standardized methodology would be methodologically more robust compared to alternative strategies. Standardization can create winners and losers^70,71^, and the final standardized methodology may better reflect the institutional power of those advocating for it^72,73^ rather than its scientific rigor. Meanwhile, even if these challenges are overcome but the desired data is part of a longer time series, then previously collected data cannot be included in prospective harmonization^74^. It is also not always possible to anticipate future data needs, and as the history of national accounts can attest to, in the worst case scenario, the same variable is used to measure different concepts over time^75–77^. Finally, there are often real world constraints which limit the utility of prospective data harmonization. For instance while participants in the European Influenza Surveillance Scheme coordinate to monitor seasonal influenza strains, differences in health care and health insurance systems limit the extent to which there can be congruence in the output data^78^.

To tie back to the previous section, recall that we introduced the idea of stringent or flexible harmonization as a way to conceptually understand data harmonization. Note that conceptual understandings of data harmonization as stringent or flexible are orthogonal to procedural understandings of data harmonization as retrospective or prospective. For example, it is entirely possible for data to be both retrospective and stringently harmonized. Our harmonization of 8 PHSM datasets can be so characterized insofar as all data on government policies from the 7 datasets external to the CoronaNet dataset are recoded based on the original (government or news) source into the CoronaNet taxonomy, making their functionally identical to those already captured by the CoronaNet dataset. It is also entirely possible for data to be harmonized both prospectively and flexibly insofar as data scientists agree on a core set of variables or measures to harmonize but allow some flexibility in how it is collected across studies^34^.

Separate from when data is harmonized (i.e. retrospectively or prospectively) is how it is harmonized. While there are many different ways of harmonizing data, they can broadly be described in two archetypes, **merging** and **mapping**^32^. Merging entails developing a single global taxonomy or ontology that can encompass taxonomies or ontologies across disparate datasets. The benefit of this approach is that it contains all possible information across disparate datasets but the drawback is that it may be difficult or time-consuming to develop and the resulting ontology may be unwieldy or impracticable to use. For instance, suppose ontology A captures information on curfews, restrictions of public gatherings over 100 people and travel bans. Meanwhile ontology B captures information on lockdowns, restrictions of public gatherings over 200 people and travel bans. One way to harmonize the different datasets which use either ontologies A or B would then to develop a new ontology X which captures information about curfews, lockdowns, restrictions of public gatherings over 100 people, restrictions of public gatherings over 200 people and travel bans. Another way to would be to develop a new ontology Y which captures information about (i) curfews, lockdowns, restrictions of public gatherings and travel bans and (ii) how many people are restricted from gathering publicly (e.g. 100 or 200). Both ontologies X and Y would be examples of developing a global ontology to harmonize ontologies A and B.

Mapping meanwhile creates a set of rules to relate different taxonomies or ontologies to each other. A benefit of this approach is that original information in a given dataset can be preserved but a drawback is that mappings can become complicated if many-to-one mappings are necessary or one-to-one mappings are not possible. Suppose ontologies A and B are the same as in the example for merging above. While it would be trivial to map information travel bans from ontology A to dataset B or vice versa, mapping the other policy measures is more difficult. For instance, while it would be possible to map ‘restrictions on public gatherings with more than 200 people’ to ‘restrictions on public gatherings with more than 100 people’ (since the former is a subset of the latter), it would not be easily doable to directly map the reverse. Meanwhile, though lockdowns and curfews are similar to each other, if one sticks to the strict definition that lockdowns compel people to stay at home at all times except to procure food while curfews compel people to stay at home only during certain times of day, mapping lockdowns and curfews to each other would be impossible regardless of the directionality (ontology A to B or B to A). Mappings could be possible however, if one relaxed or created a new policy category of ‘lockdowns or curfews’ which captures policies that compel people to stay at home at all times except to procure food or during certain times of day.

Merging and mapping are orthogonal to prospective and retrospective harmonization in that they can be mixed and matched together. With regards to flexible and stringent harmonization, in a practical sense, both merging and mapping can be understood as strategies to implement flexible harmonization insofar as they entail resolving differences in how data is measured, an issue which stringent harmonization by definition sidesteps. In a more academic sense however, merging and/or mapping does take place, though likely more informally, even for stringent harmonization insofar as data scientists with disparate analytical needs must resolve their differences in order to cooperate on a stringent harmonization methodology.

### Implementation Steps

**Fig. 1:**
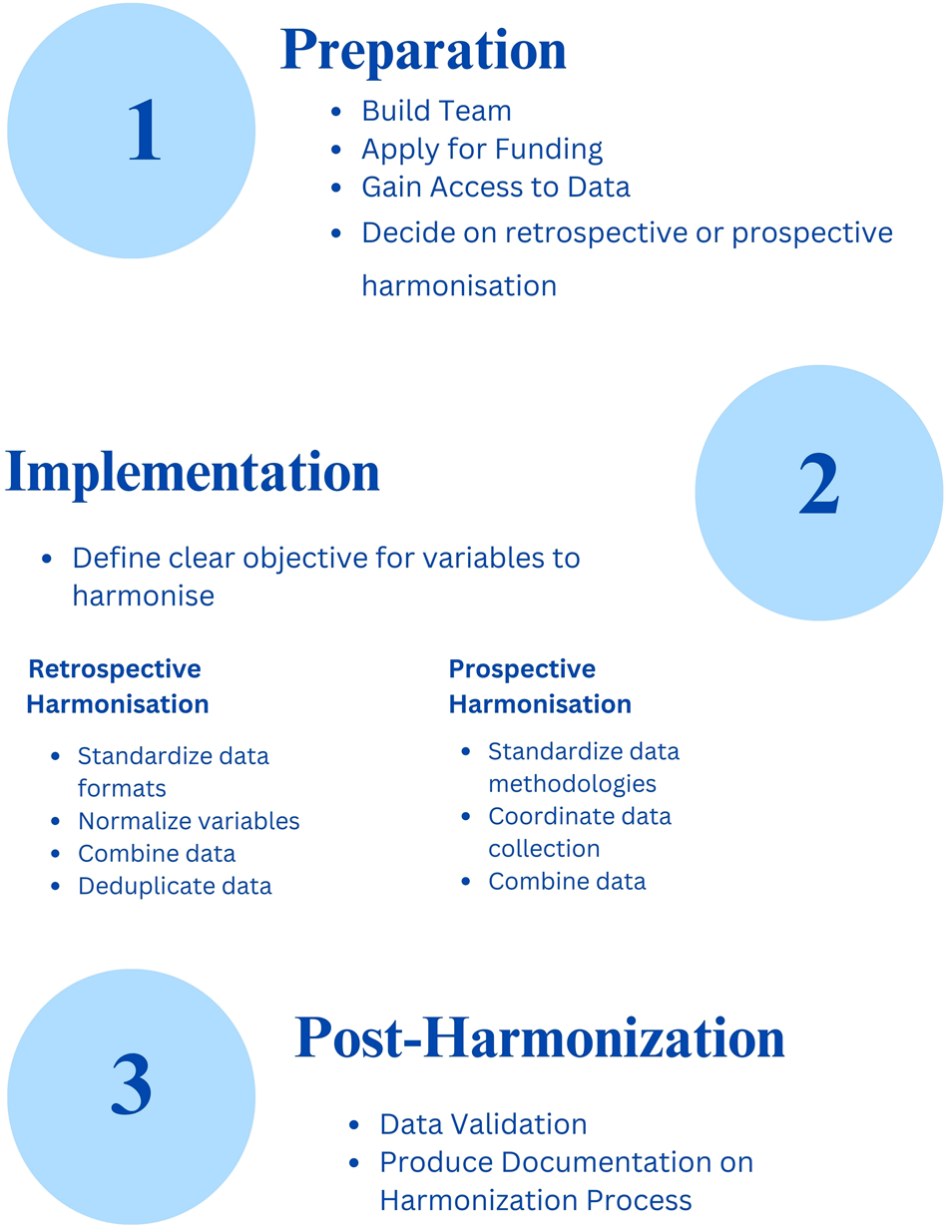
General Steps for Data Harmonization.

In Fig. 1, we outline the basic steps for data harmonization. We note however, that there can be a great deal of variance in terms of what steps are necessary depending on the field. For instance, while deduplication for retrospective data harmonization may be an important issue for datasets on overlapping geographical maps, it is less likely to be an issue when combining datasets on different samples of patients using medical data. Moreover, while the steps below are presented linearly, in practice data harmonization is an iterative process with much back and forth between different steps, which echoes Fortier *et al*.'s^39^ caveat for their data harmonization guidelines for epidemiological data (indeed, note that the order of the steps presented in Fortier *et al*.^39^ are different from those presented in Fortier *et al*.^20^). Researchers should consult field-specific guidelines, to the extent that they exist, for detailed procedural steps most relevant for their purposes. Note while specific to epidemiological data and survey data respectively, data scientists outside of these fields may also find the guidelines developed by Fortier *et al*.^20^ (Box 1 and 2 in particular provides a succinct overview) and SRC^31^ to be helpful for a more detailed step-by-step account of retrospective data harmonization protocols.

#### Step 1 Preparatory stage

The chicken and egg aspect of data harmonization is especially pronounced in the preparatory phase. To begin with, the size and desired skill set for building a data harmonization team is to some extent dependent on how many datasets there are to harmonize and what kind of processing will need to be done in the implementation stage. Meanwhile the amount of funding that may be necessary to complete data harmonization can also be dependent on a number of factors, including the number of datasets involved, the necessity of purchasing dataset(s), the cost of storing or securing dataset(s), and the length of time and effort projected for harmonizing data during the implementation stage (which itself is a function of the quality and type of data desired for harmonization)^39^. Moreover, gaining access to data is not necessarily only a function of financial resources, but can also be a function of legal and ethical considerations, including data privacy. With regards to medical data especially, data availability can range from more or less immediate access to several months or a year or more^39^. During this stage, data scientists must decide whether, conditional on the decision to proceed with data harmonization (as opposed to e.g. original data collection or pulling the plug), they will pursue retrospective or prospective data harmonization. The decision to do one or the other will depend on what data can be accessed as well as the time and (human and financial) resources available for harmonizing data.

#### Step 2 Implementation stage

Across both retrospective and prospective harmonization, data scientists must first define which variables they wish to harmonize. To make this decision, they must consider both the ultimate goal of the data harmonization (e.g. to conduct a theory-driven approach in order to test relationships between a given set of variables or to conduct a data-driven approach in order to discover relationships between a broad set of variables^51^), what data is available for harmonization, and the acceptable level of harmonization. Note, in some fields, the term “DataSchema” is increasingly used to refer to the core variables that are ultimately selected for harmonization^20,79–81^.

Deciding on retrospective harmonization means making comparable already existing and collected but heterogeneous data, some or none of which a given data scientist team may have themselves previously collected. To do so, data scientists must ensure that differences in technical formats (data syntax) and structure (data structure) of different datasets are resolved. They must further make comparable the meaning of different measures (data semantics). Once these dimensions are harmonized, then the different datasets can be combined and deduplicated if necessary.

Meanwhile, deciding on prospective harmonization means coordinating on the same procedure for collecting data across multiple instances of data collection. This can range from e.g. different data scientists coordinating to collect data in different countries more or less simultaneously, or the same data scientists collecting data on different populations across time or some combination thereof. The first step here is to harmonize data methodologies across different data collection efforts. Note that this does not mean that reconciling data syntax, structure and semantics are no longer relevant but that these issues have been subsumed within the step of harmonizing data methodologies. In other words, what retrospective harmonization makes explicit insofar as datasets with many different structures and syntaxes are in existence and must be dealt with, prospective harmonization makes implicit insofar as different data syntaxes and structures are considered when deciding between data methodologies but never come into being in the form of an actualized dataset^75^. As discussed in our section on prospective harmonization above however, deciding on a harmonized data methodology is often not a trivial task and in many ways can be more difficult than retrospective harmonization. Following this, the next step in prospective harmonization comes from coordinating data collection and ensuring that standard protocols are indeed being followed. Once the data is collected, they must then be combined. In theory, if standard methodologies have been followed, then the processing stage here is minimal compared to retrospective harmonization.

While it is beyond the scope of this article to go into specifics, we note that the methods for rendering data comparable or compatible vary widely depending on the type of data being harmonized. For example, common methods for harmonizing textual data include text preprocessing, Natural Language Preprocessing, machine learning and deep learning^82^. Meanwhile, epidemiological or health data are often processed using algorithmic transformation, simple calibration models, standardization models, latent variable models or multiple imputation models^20^. Readers should consult field-specific guidelines or existing harmonization efforts for more detail about relevant techniques in their respective areas of interest.

#### Step 3 Post-Harmonization

While the different steps laid out in Step 2 suggests that in theory, the end result should be a harmonized dataset where measures from different variables have been made compatible and comparable for analysis, without instituting a **data validation** procedure, it is impossible to assess whether such a result was empirically achieved. Appropriate validation procedures will differ depending on the nature of the underlying data and can range the gamut from replicating and checking the harmonization procedure on a sub-sample of the data to employing machine-learning based solutions; consulting field specific guidance will be especially helpful in this case.

Correspondingly, though the concrete points laid out in Step 2 suggest a straightforward process for data harmonization, in practice, it can involve variegated and nuanced methodological decisions (common to both types of harmonization) which should be **transparently documented** for future data users. For example, making the choice to harmonize on a certain semantic understanding of a word, (e.g. defining COVID-19 lockdowns as policies which restrict people to their homes to reduce the spread of the COVID-19 disease) means forgoing other semantic understandings (e.g., defining COVID-19 lockdowns as the closure of both public and private institutions to reduce the spread of the COVID-19 disease). Making choices on not only semantic harmonization transparent, but on all choices made at different levels of the data harmonization process help users better understand how the resulting harmonized dataset can be used and what its benefits and drawbacks are. It can further help ensure that they accord to the Findable, Accessible, Interoperable and Reusable (FAIR) standards^51,83^, which is increasingly being adopted by different fields to encourage data sharing.

## What tradeoffs should be considered when harmonizing data?

While the section above provides a foundation for understanding what data harmonization is and how it might be achieved, this section encourages readers to think about the tradeoffs of pursuing it in the first place relative to original data collection. Axiomatically, without original data, there is nothing to harmonize. Conversely however, the existence of original data is not itself a sufficient condition for pursuing data harmonization. That is, even if data harmonization is technically possible, ultimately, researchers should not lose sight of the fact that the end goal is to produce a complete and clean set of variables which best suit their analytical or research needs, for which harmonization may not necessarily be best suited.

However, given the myriad of dimensions there are to consider when working toward this goal, including the volume of data collected, the representability or generalizability of the resultant dataset, as well as its accessibility and transparency, to say nothing of the resources required to do so, it can be quite complicated to sift through these relative tradeoffs. To help readers think through them, we organize this section in terms of a series of questions about the relative benefits, losses, limits, and requisite (cooperative) resources associated with data harmonization compared to original data collection.

### What can be gained from data harmonization?

In increasing the volume or heterogeneity of data, harmonized data can be an improvement over original data in a number of different ways.

For one, using harmonized data can increase the statistical power of subsequent analyses compared to those done on individual datasets^12,84^. Deriving reasonable estimates of how data harmonization can forward a desired analyses either in terms of data completeness, data quality or data validity can help researchers assess the relative value of engaging in data harmonization. When it is not possible to derive such estimates beforehand, we suggest that researchers conduct pilot studies in order to gain a more concrete sense of what can potentially be gained.

For another, harmonizing data can allow researchers to assess the generalizability or transportability of a given finding^85,86^. Note that while the two terms are closely related, generalizability refers to whether causal effects can travel from the sample to the population under question (e.g. from a survey of students in a given school to all students in the school) while transportability refers to whether causal effects can travel to (at least partly) external populations (e.g. from a survey of students in a given school to students in other schools)^87^. Note, transportability analysis has been found to be possible even when harmonizing observational and random control trial (RCT) data^88^.

Moreover, data harmonization can also increase the possibility of using methods which rely on increased sample size and data variation. For instance, it can facilitate the exploration of interactive effects of variables within the data^89,90^ or make possible subgroup analyses to identify variation in within-sample effects^91,92^. Note, subgroup analysis divides a sample into different subsets based on an observable characteristic (e.g. gender, age) in order to investigate whether treatment effects differ among them. They have a number of uses, including helping to identify the most impactful treatment from a set of possible treatments, characterizing an ideal treatment for a given observation, addressing concerns about replicability and helping researchers identify likely causal mechanisms for designing future experiments^93^[pg.2], so long as reporting is transparent^94^ and sample sizes are sufficient (or appropriate estimators are used)^95^.

Meanwhile, to the extent that the harmonization process can identify and rectify previous miscodings or biases in the original data collection process, it can improve the overall data quality. In our experience with harmonizing data on COVID-19 government responses for example, we identified and rectified a substantial amount of data quality issues in the original data, especially with regards to missing data and missing sources^29^.

Finally, data harmonization may also greatly increase access to original datasets which previously were not widely accessible to the public. This particular issue is salient not only for medical data (given the importance of safeguarding patient privacy)^96,97^ but can affect other fields as well^98^, especially if data sharing is not the norm or few resources exist to support it^99,100^. Even data that are technically available for public use may be functionally unavailable if documentation is poor. The data harmonization process can help address this issue when it can produce cogent documentation of how the data was gathered or a third-party evaluation of the quality of the underlying data^101,102^.

### What can be lost from data harmonization?

Although this commentary was motivated by the observation that data harmonization can help address the bottleneck of data production, this does not mean that harmonized data is is always the dominant strategy for conducting data analyses. Especially given trends toward increased quantification and datification of natural and social phenomena^103^, losing sight of what the data is actually measuring or how well it is doing so is all too easy^104^. This section in particular focuses on exploring what conceptual diversity may be lost because of the harmonization process as well as how well harmonized data can operationalize relevant concepts relative to original data collection.

Fundamentally, the creation of more harmonized or standardized data can come at the expense of conceptual diversity or complexity^105^[pg. 11-12]. The existence of different datasets in a given field often underscores the possibility of having distinct, yet valid conceptualizations and operationalizations of a given topic. Harmonizing different datasets may increase the internal coherence of a given concept at the expense of minimizing real and potentially important diversity in theoretical approaches toward a given topic. While this is arguably a greater problem with prospective harmonization given that alternative ways to collect data will not be realized to begin with, it can also be a problem for retrospective harmonization. That is, although the original datasets will still exist after being retrospectively harmonized, they may nevertheless fall into disuse or irrelevance if the subsequently harmonized data and/or the ontology that it represents comes to dominate analyses in the field. Moreover, there is also the risk that researchers may invest significant time and resources in creating standardized measures only to see them become obsolete in the face of rapid evolution in technology^105^[pg. 11-12]. Researchers bent on creating harmonized or standardized measures at all costs should be cognizant of the risk of pandering to the lowest common denominator to achieve comparability and thus losing “important meta data or disconnection from local meanings and circumstances”^35^. If after conducting an assessment of what may be lost from data harmonization, the researcher decides to proceed, making these tradeoffs transparent for the research community overall can contribute to the rigor of analyses conducted in that field.

Assuming data harmonization is in fact pursued, data scientists must then grapple with how well data harmonization can produce variables that accurately operationalize a given concept. Frequently, data scientists must make trade-offs between the quantity of data that can be harmonized and the precision with which the harmonized data operationalizes the desired underlying concepts^69,106–109^. Torres-Espin and Ferguson articulate this idea as the harmonization-information tradeoff^51^ which states that “the level of granularity in harmonizing data determines the amount of information lost.” While they focus specifically on biomedical data in their analysis, their insights can be broadly applicable to harmonization across fields. Factors which affect this tradeoff include the (i) availability of of timely and relevant data (a particular issue in fields without established norms for data sharing) (ii) the quality of data collected (a particular issue when data collection methodology is not transparent or accessible) (iii) and the comparability, and therefore the ultimate potential for harmonizing the underlying data (common across all fields). To illustrate these tradeoffs through COVID-19 school restriction policies, note the OxCGRT dataset^33^ uses an ordinal variables to capture this information as follows:

- 0 - no measures
- 1 - recommend closing or all schools open with alterations resulting in significant differences compared to non-COVID-19 operations
- 2 - require closing (only some levels of categories, e.g. just high school or just public schools)
- 3 - require closing all levels.

Meanwhile, as shown in Table 1, the CoronaNet taxonomy captures information on which type of school is targeted (e.g. preschool, high school) in its type_sub_cat field, what level of restriction is applied (e.g. allowed to open with no conditions or some conditions) in its institution_status field, and if a school is allowed to open with restrictions, what those restrictions are (e.g. limited number of students, cleaning and sanitary procedures) in its institution_conditions field.

**Table 1:** CoronaNet taxonomy for capturing COVID-19 schools policies.

[type]	[type_sub_cat]	[institution_status]	[institution_conditions]
**Closure and Regulation of Schools** *Government policy which regulates educational establishments in a country*	• Preschool or childcare facilities (generally for children ages 5 and below) • Primary Schools (generally for children between ages 5 and 10) • Secondary Schools (generally for children between ages 10 and 18) • Higher education institutions (i.e. degree granting institutions)	• [type_sub_cat] allowed to open with no conditions • [type_sub_cat] allowed to open with conditions • [type_sub_cat] is closed/locked down	• Number of people on the school premises are limited (e.g. only 50 people allowed on school premises) [Text Entry] • Types of people on school premises are limited (e.g. no parents) [Text Entry] • Physical classroom hours or meeting times reduced (e.g. classes only meet in the morning; classes meet every other day) [Text Entry] • Negative COVID-19 Test • Full remote/distance learning (e.g. All teachers must tele-teach.) [Text Entry] • Partial remote/distance learning (e.g. Teachers with certain health conditions can tele-teach) [Text Entry] • Special provisions exist for how teaching is done which applies to all teachers [Text Entry] • Special provisions exist for how teaching is done which applies to some teachers [Text Entry] • Special provisions for all students in a school (e.g. students in primary school do not have to social distance) [Text Entry] • Special provisions exist for some students in a school (e.g. students living with essential workers can attend school while others must stay home) [Text Entry] • School event cancelled or postponed [Text Entry] • Other conditions not listed above (please provide detail in the text entry) [Text Entry] • Temperature Checks • Health Certificate • Health Questionnaire • Other Health Monitoring [Text entry]

Full taxonomy table available here: https://www.coronanet-project.org/taxonomy.html.

Both datasets are limited by data availability and data quality issues insofar as they collected and published information on COVID-19 policies in real time with limited resources in order to respond to the emergency situation. In short, neither dataset had full geographic or time coverage of school policies nor could either guarantee completely clean data at the time of its release. In terms of comparability, harmonizing the two datasets into the OxCGRT taxonomy would mean sacrificing the granularity captured in the CoronaNet taxonomy but would allow as much data as possible from the CoronaNet dataset to be retained. Meanwhile, harmonizing them as given into the CoronaNet taxonomy would mean losing the data coded in the ‘1’ and ‘2’ categories documented in the OxCGRT taxonomy but with the benefit that the CoronaNet’s granularity can be retained. Our harmonization effort of these data, as part of our larger harmonization of 8 PHSM datasets overall, sidesteps this issue by recoding the original sources for COVID-19 policies documented by the OxCGRT dataset into the CoronaNet taxonomy, which allows us to retain both the granularity of the CoronaNet data without losing any data from the OxCGRT dataset. By pursuing this strategy however, we are confronted with additional tradeoffs in time and resources for completing harmonization; digging back into original sources takes substantially more effort than taking the data from the OxCGRT dataset at face value^29^. Generally however, the harmonization-information tradeoff is a hard constraint in many fields because of the nature of the underlying data or data collection methodology. For instance, it is generally not possible to conduct a survey or take biomedical measurements in a way that perfectly replicates the conditions present in the original study as e.g. the same people may not be available for study or may be experiencing different life situations compared to the original study.

Meanwhile, though dealing with issues of data comparability is the bread and butter of data harmonization, ensuring conceptual comparability is a particular issue when harmonizing data across different social contexts. Indeed, how a dataset is constructed, i.e., its syntax, is often “dependent upon the units and scale of measurement within each social system”^110^[pg. 40]. As Boyden and Walnicki find^35^, even when different datasets contain similar information about household wealth, standardizing these measures across different survey rounds and national contexts was not possible, often due to semantic differences. They themselves split the difference by creating a multidimensional wealth index instead, which allows for the inclusion of more observations at the cost of less precision in the operationalization of the original measures. Meanwhile, measurement differences can also be a function of historical timing even if all other dimensions of the data collection process are held constant if each study tailors itself be maximally valid for a given period^105^[pg 14]. In short, while data harmonization can often be of great value when it combines datasets across different geographical areas or time, researchers must account for the (social) contexts in which such datasets were conceptualized and gathered to ensure functional, linguistic and cultural equivalency of the desired variables^111^.

What should be avoided at all costs is combining datasets that ultimately measure different concepts, leading to false or inappropriate equivalences and nonsensical measures. Given its failure to reconcile data into a compatible or comparable form, in some sense, such data cannot be considered to be harmonized at all. Regardless of nomenclature, given that such data is unfortunately produced with some frequency^43^, it warrants a mention here. Careful exploration and comprehension of the underlying datasets to make sure they are inferentially equivalent, and thus appropriate to harmonize is imperative to avoid such outcomes^105^[pg 17-18].

Original data collection, by contrast, will virtually always allow data scientists to operationalize a given concept at least as or more precisely than harmonized data. However, original data collection may not always be possible. This can occur for any number of reasons, including (i) difficulty in identifying data to collect in a timely manner^112^. E.g., researchers studying rare diseases may find it virtually impossible to identify large samples of relevant patients quickly^113^. (ii) Data collection may be prohibitively expensive even if the sample size needed is relatively small. E.g., studies that rely on neuroimaging often use data from less than 50 individuals in part because of the high material costs of MRI imaging^12^. Relatedly (iii) the number of observations needed may be prohibitively large, which may make original data collection unfeasible due to insufficient resources or lack of requisite authority. E.g., although (geo-)data of human populations is a lynch pin of social science research, many countries lack detailed census data due to insufficient resources. Meanwhile global census data is not possible due to both cost and jurisdictional constraints^22,23^.

### What are the limits of data harmonization?

Harmonizing data is rarely equivalent to building a universally complete or coherent dataset. That is, putting aside the issue of what may be lost in creating a harmonized dataset, the harmonized dataset itself often has limitations that should be acknowledged and evaluated. Here we consider limits in the volume of data that can be harmonized as well as the subsequent validity of harmonized data.

One real limitation is that observations included in the harmonized datasets are often only a subset of what could theoretically be collected. That is, if the underlying datasets do not themselves contain the desired data, then the harmonized data will also face the same data limitations. For example, in our harmonization of 8 COVID-19 PHSM datasets, we found that all datasets likely systematically underreport data from countries that are not in North America or Europe. While data harmonization can increase the absolute number of data points for these other countries, it cannot rectify its relative paucity to North American or European ones^29^.

Despite the importance of reporting the scope of one’s data harmonization efforts, researchers do not appear to consistently report this information^56,57,112^, with this being a particular problem some fields^114^ or subfields over time^115^. Meanwhile, other fields have made progress on providing ready-made tools and resources for researchers to perform such an evaluation^116^. Reporting on the limitations of data harmonization is important for helping a given field identify research gaps or giving researchers proper context for using a harmonized dataset.

Moreover, the richness of information that is ultimately harmonized may often be shallow or superficial. The field of epidemiology in particular appears to be “filled with large but information-poor (shallow) data sets that feature a small number of variables with high numbers of subjects”, but such a characterization could very well be applicable to any field which employs methods which “emphasize[s] high sample sizes to boost statistical power”^51^.

Meanwhile, the data harmonization process may propagate existing errors from original datasets^117^ or generate new ones during the data harmonization process which can limit the validity of the subsequent data. Indeed, measurement error, that is the difference between the measured value and its true value, can be found even when harmonizing very similar data. For instance, MRI diffusion scans display variation even when using the same scanner (to say nothing of across-scanner variability)^12,118^. More generally, measurement error can occur for any number of reasons, from differences in technicians, materials or instrumentation (often known as batch effects) when dealing with medical data^119^ to those introduced by interviewers, respondents, the questionnaire or data collection methodology in survey data^120^.

Strategies to address such measurement errors head on may range from analog to technical. Analog methods include (i) recruiting larger sample sizes in the underlying data to more closely approximate the population-wide distribution or (ii), in the case of human subject datasets, recruiting a subset of subjects who can travel to multi-site locations to calibrate measurement errors^26,121^. Meanwhile, other researchers have proposed statistical techniques to account for measurement errors, from simpler strategies like pre-processing^122^ or outlier detection methods^123^ to more complex model-based techniques, like e.g. linear or deep learning models, which tend to be quite specific to different fields and datasets^123–127^.

While the above strategies can help address measurement error issues, its ability to do so is conditional on (i) the number of datasets harmonized (the more the better) and (ii) the extent to which a systematic error for a given dataset is random at the level of the dataset (i.e. the same type of error is not made in all individual datsets). In the worst case, the number of harmonized datasets are few and all exhibit the same types of error, which can compound the errors underlying the original dataset. However, even if the above conditions are fulfilled, it would still desirable to reduce measurement errors in order to improve the overall precision of subsequent analyses conducted with the harmonized data.

### What resources are available for harmonizing data?

Data harmonization, like all data processing efforts including original data collection, require substantial time and resources to conduct. In this section, we provide an overview of resources particular to the data harmonization process, specifically cooperative resources and existing data harmonization tools.

Given that data harmonization often requires involves working with data collected by disparate people or organizations, the importance of cooperation for its success is particularly pronounced. Indeed, across the numerous harmonization efforts we have surveyed, virtually all emphasize the importance of cooperation and coordination among different partners. Given that, ultimately, data harmonization entails translating each dataset to speak the same ‘language’, exchanges between those engaged in harmonization and those who collected the underlying data is useful for both resolving confusion or misunderstandings about different taxonomies, ontologies or methodologies and increasing the capacity for piloting data harmonization efforts in a timely manner. That being said, researchers have also underscored that maintaining such cooperation can itself be a resource-intensive undertaking^52,128^.

While the form of communication and cooperation across different harmonization efforts will necessarily be idiosyncratic, given its importance to the success of data harmonization, accounting for these potential cooperative resources is important for evaluating the subsequent feasibility of data harmonization. Furthermore, providing some documentation in this regard can be helpful for evaluating the quality of the subsequently harmonized data and can increasing the transparency of the data generating process.

Meanwhile, more and more technical tools for harmonization are being made available to help data scientists, especially in the natural sciences, engage in data harmonization without having to reinvent the wheel. Some examples of tools to aid in data harmonization include: the DataHarmonizer, a standardized browser-based spreadsheet editor which is geared toward genomics data^129^ and HarmonizeR, an R package which makes available an algorithm can deal with missing data in omics datasets^130^. Researchers, especially in epidemiology, may further benefit from making use Rmonize^131^, an R package which provides functions to support retrospective data harmonization, evaluation and documentation based on the guidelines developed by Fortier *et al*.^20^. Meanwhile, platforms include those developed by the Predicting OptimaL cAncer RehabIlitation and Supportive care (POLARIS) study for harmonizing individual patient data^132^ as well as the Phenopolis platform for harmonizing data on genes phenotypes^133^. To the extent that such tools or platforms are used, they can (i) help harmonize or standardize the harmonization process itself and/or (ii) reduce start-up costs to those seeking to engage in data harmonization. As with data harmonization more generally, thoughtful consideration as to whether a given tool or platform best suits one’s research or data needs is advisable before using such tools. Admittedly however, if at some point there is a critical mass of studies which use a given tool or platform, the network benefits of using that tool may well outweigh its potential downsides.

## Discussion

Implicit in this commentary is the idea that statistical measurement is not a strict reflection of reality but rather a construction of it^75^. In weaving together similar concepts that are nevertheless operationalized and/or measured differently across different datasets, data scientists are not only shaping how useful the data can be for their own analysis, but given the substantial time and resources necessary, are also likely shaping it for others who may use it in the future. To that end, data scientists must balance a variety of tradeoffs when creating their finalized harmonized dataset, including the subsequent size of the data, its internal and external validity as well as its generalizability and quality. Clear understanding and documentation of these tradeoffs can help researchers tailor their data harmonization efforts to best suit their analytical needs as well as help others evaluate these efforts.

In our commentary, we have sought to provide general primer and guidance on these issues by first providing an overview of what data harmonization is and how it can be broadly implemented. We then provide a discussion of the various complexities and challenges to consider when creating, using or evaluating harmonized data. The limitation of providing such a general focus naturally, is that it cannot provide more detailed instructions for how to implement data harmonization for specific fields or datasets. Similarly, because of its general nature, this commentary is also unable to provide specific recommendations as to how to deal with various trade-offs when harmonizing data. Field-specific, transparently documented work will be crucial to consult in these cases. However, we hope that by raising these general points of consideration, which we derive from both our first-hand experience with harmonizing data on COVID-19 PHSM as well as an overview of existing work in data harmonization from a wide variety of fields, our primer can nevertheless help spread awareness of what issues creators and evaluators of data harmonization should pay attention to.
